# Advancement of epigenetics in stroke

**DOI:** 10.3389/fnins.2022.981726

**Published:** 2022-10-13

**Authors:** Jianhua Peng, Dipritu Ghosh, Fan Zhang, Lei Yang, Jinpeng Wu, Jinwei Pang, Lifang Zhang, Shigang Yin, Yong Jiang

**Affiliations:** ^1^Department of Neurosurgery, The Affiliated Hospital of Southwest Medical University, Luzhou, China; ^2^Laboratory of Neurological Diseases and Brain Function, The Affiliated Hospital of Southwest Medical University, Luzhou, China; ^3^Institute of Epigenetics and Brain Science, Southwest Medical University, Luzhou, China; ^4^Academician (Expert) Workstation of Sichuan Province, The Affiliated Hospital of Southwest Medical University, Luzhou, China; ^5^Sichuan Clinical Research Center for Neurosurgery, The Affiliated Hospital of Southwest Medical University, Luzhou, China

**Keywords:** stroke, epigenetic, histone, DNA/RNA modification, neuroinflammation

## Abstract

A wide plethora of intervention procedures, tissue plasminogen activators, mechanical thrombectomy, and several neuroprotective drugs were reported in stroke research over the last decennium. However, against this vivid background of newly emerging pieces of evidence, there is little to no advancement in the overall functional outcomes. With the advancement of epigenetic tools and technologies associated with intervention medicine, stroke research has entered a new fertile. The stroke involves an overabundance of inflammatory responses arising in part due to the body’s immune response to brain injury. Neuroinflammation contributes to significant neuronal cell death and the development of functional impairment and even death in stroke patients. Recent studies have demonstrated that epigenetics plays a key role in post-stroke conditions, leading to inflammatory responses and alteration of the microenvironment within the injured tissue. In this review, we summarize the progress of epigenetics which provides an overview of recent advancements on the emerging key role of secondary brain injury in stroke. We also discuss potential epigenetic therapies related to clinical practice.

## Introduction

Stroke is one of the main leading causes of death and the first leading cause of disability worldwide (Avan et al., 2019; Collaborators, 2019). Hemorrhagic stroke, including intracerebral hemorrhage (ICH) and subarachnoid hemorrhage (SAH), happens when a blood vessel in the brain bursts or when brain tissue starts to bleed. On the other hand, ischemic stroke (IS) directly results from the disruption of blood supply to the brain and constitutes approximately 85% of all known cases of stroke. After the stroke, injured brain parenchyma initiates biochemical cascades, which include energy failure, ionic pump failure, oxidative damage, cell death, and inflammation, eventually leading to irreversible brain damage (Iglesias-Rey et al., 2022). Additionally, patients surviving stroke may suffer from functional disabilities that might require temporary or lifelong assistance (Aslanyan et al., 2003). Thus, understanding stroke at the molecular level will help researchers to produce key therapeutic strategies to minimize secondary injuries and promotion of neuroprotection associated with stroke (Saini et al., 2021).

Over the past few decades, researchers have advanced in our understanding of the epigenetic mechanisms involved in the central nervous system (CNS) and its role in neuropsychiatric disorders (Szyf, 2015). These epigenetic-related findings also offer the important translational potential for stroke research. Thus, fully understanding the role of epigenetic regulators in the stroke process is crucial to harness the potential of epigenetic therapies. Here, we review three epigenetic mechanisms involved in secondary brain injuries post-stoke: histone modification, DNA-methylation, and RNA modifications. We also discuss the relevant clinical treatment targeting epigenetics and summarize future advancements in this field.

## Etiology

The mechanism and pathophysiology involved in ischemic stroke and hemorrhagic stroke are quite different but with some overlap. Two major mechanisms responsible for acute ischemic stroke (AIS) are thromboembolism and hemodynamic failure. Embolism, more precisely cardio-embolism, has been demonstrated to produce 20 to 30% of all ischemic strokes (Kolominsky-Rabas et al., 2001; Kamel and Healey, 2017). Risk factors associated with cardio-embolism include atrial fibrillation (Kamel et al., 2016), systolic heart failure (Go et al., 2001), acute myocardial infarction (Putaala and Nieminen, 2018), patent foramen ovale (Gottdiener et al., 1983), aortic arch atheroma (Witt et al., 2006), prosthetic heart valves (Cannegieter et al., 1994) and infective endocarditis (Kim and Kim, 2018). Large vessel atherosclerosis (LVA) is another main contributor to ischemic stroke. LVA accounts for nearly 15 to 20% of all ischemic strokes. In addition, small vessel occlusion is also a culprit that can be diagnosed in approximately 25% of patients with ischemic stroke (Grau et al., 2001). Hemorrhagic stroke, on the other hand, has a well-established relationship with traumatic brain injury (TBI) (Chen et al., 2011), cerebral aneurysm (Nieuwkamp et al., 2009), anti-thrombolytic therapy (Puy et al., 2022), hypertension (Wan et al., 2022), and other cerebrovascular diseases.

## Pathophysiology

Under injured conditions, several molecules can gain access to the cytoplasm of the cell and leak from the dying cells into the extracellular environment. These spilled substances such as DNA are not only manifested as changes in expression but also in their own structures, these changes will gather a series of complex secondary pathophysiological processes (Eser Ocak et al., 2020; Gamdzyk et al., 2020). The pathophysiology involving stroke is quite complex and involves various cascade processes, which include: loss of cellular homeostasis, energy failure, metabolic acidosis, increased intracellular Ca^2+^ levels, free-radical mediated toxicity, generation of arachidonic acid products, cytokine-mediated cytotoxicity, complement activation, apoptosis, autophagy, disruption of the blood-brain barrier (BBB), activation of glial cells and infiltration of leukocytes (Lu et al., 2021; Qiu et al., 2021; Ye et al., 2021; Peng et al., 2022). The mechanism involved in both ischemic and hemorrhagic stroke produces significant cerebral hypoperfusion leading to an increase in anaerobic metabolism and eventually, lactic acidosis which in turn sequentially causes astrocyte demise and an increase in neuroinflammatory cytokines.

**FIGURE 1:**
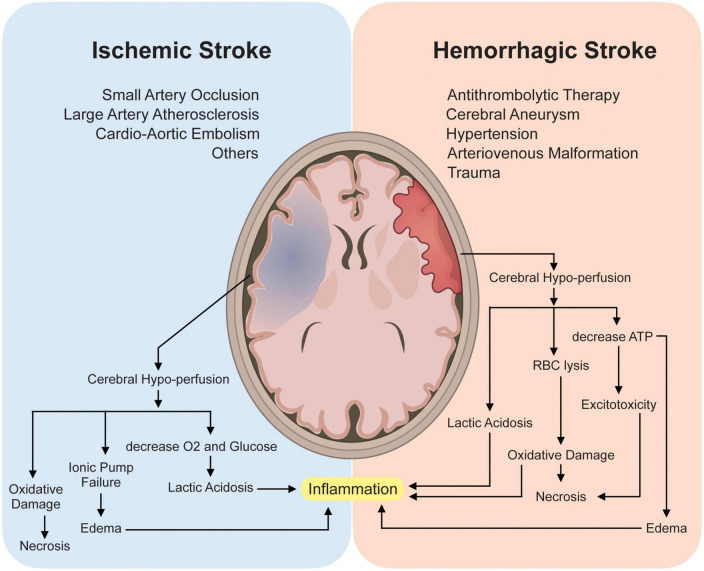
Pathophysiology and Mechanism involved in Ischemic and Hemorrhagic stroke. Briefly, mechanisms involved in both ischemic and hemorrhagic stroke involve cerebral hypo-perfusion leading to oxygen (O_2_) deprivation causing an increase in anaerobic metabolism and eventually lactic acidosis which sequentially causes astrocyte demise and an increase in neuroinflammatory cytokines thus promoting neuroinflammation. Subsequently, cerebral hypo-perfusion can also cause malfunction of the ionic pump causing potassium ions (K^+^) efflux, sodium and calcium (Na^+^ and Ca^2+^ respectively) influx into the neuronal cells and adenosine triphosphate (ATP) depletion causing excitotoxicity, edema, and eventually led to necrosis. Red blood cell lysis can further cause oxidative damage which further supports necrosis.

Neuroinflammation has been recognized as one of the main culprits in promoting further insults in post-stroke conditions, however, they also play a beneficial role in functional recovery (Bourhy et al., 2022). Similarly, a decrease in cerebral hypo-perfusion can also produce malfunction of the ionic pump causing potassium ions (K^+^) efflux and sodium, calcium (Na^+^ and Ca^2+^, respectively) influx into the neuronal cells and adenosine triphosphate (ATP) depletion causing excitotoxicity, edema, and eventually led to necrosis (Zhang et al., 2021; Zhong et al., 2022). Red blood cell lysis can further cause oxidative damage which in turn further supports necrosis (Figure 1).

## Epigenetics in research frontline

**FIGURE 2:**
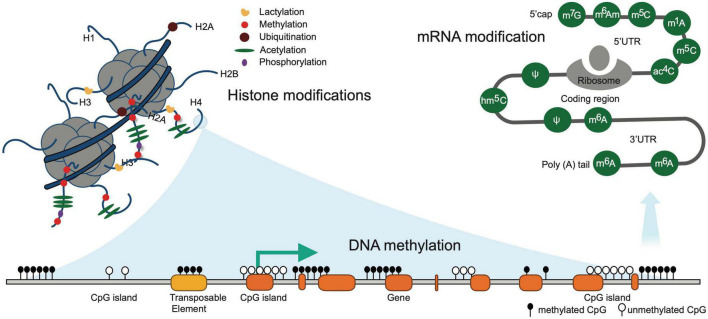
Illustrates common epigenetic modifications in stroke. DNA methylation occurring exclusively at the CpG island is associated with gene silencing and is irreversible modifications. Known histone modifications occurring at the amino-terminal tails are short-term reversible modifications. RNA modifications are the chemical alteration of the RNA molecules post transcription that alters the expression of RNA.

Epigenetics is defined as the branch of biology which studies the causal interactions between genes and their products which bring the phenotype into being. Epigenetic variation, a phenomenon that alters genome modifications without affecting DNA sequence, can affect the development of individuals (Freedman et al., 2022), cancer evolution (Nam et al., 2021), neurodegenerative disease (Corces et al., 2020), and mental disorder (Havdahl et al., 2021). In particular, dynamic epigenetic states regulate immune response and inflammation under pathological conditions (Liotti et al., 2022). Recent epigenetic studies have been demonstrated to play a key role in post-stroke conditions leading to inflammatory responses and alteration of the microenvironment within the injured tissue (Zhao et al., 2016). The current understanding and development of epigenetic tools have given the researchers a more reliable method of competitive differentiation of normal versus diseased conditions at the molecular level (Ng et al., 2018). Contemporary studies in the field of epigenetics involve Histone modification, DNA-methylation, and RNA modifications, and their association with both pre and post-stroke conditions (Figure 2).

## Histone modification

Histone is the basic protein found in the nucleus of eukaryotic cells wrapped around by 146 base pairs (bp) of DNA into a compact structure known as a nucleosome. The interaction between histone and DNA is determined by the electrical charges between them. Briefly, the histones are positively charged due to the presence of a large amount of positively charged amino acids (mainly lysine and arginine). On the other hand, DNA is negatively charged and thus interaction of positive and negative charges maintains the structural integrity of the nucleosome. Unlike DNA methylation, histone modification exclusively occurs at the amino-terminal tail protruding out of the histone subunit and is a short-term reversible modification. The amino-terminal tails are subjected to post-translational modification namely methylation, acetylation, phosphorylation, and ubiquitination (Yu et al., 2021). Post-translational modification of amino-terminal tails is associated with DNA repair, activation or repression of gene expression, telomere integrity, and the total interaction changes in response to these modifications are determined by “histone code” (Ng et al., 2018).

In humans or mammals, the immune system, especially innate immune cells, plays a decisive role in producing signals depending on the response in cerebrovascular events. The predominant innate immune cell in the CNS is microglia, along with subsidiary infiltrating myeloid cells because of the disruption of the BBB. Microglia, even under resting conditions, constantly monitor the surrounding microenvironment and act promptly per changes (Wesselingh et al., 2019). Activated microglia are subjected to altering their morphology, gene expression, and consequently undertaking their role per the changes in the microenvironment (Cherry et al., 2014). Similar to macrophages, pro-inflammatory microglia (M1) has been illustrated to up-regulate inflammatory genes namely interleukin-1 alpha/beta (IL-1α/β), interleukin-6 (IL-6), interleukin-12 (IL-12), interleukin-23 (IL-23), tumor necrosis factor-alpha (TNF-α), inducible nitric oxide synthase (iNOS) whereas the anti-inflammatory subtype (M2) has been illustrated to up-regulate neuroprotective genes such as arginase 1 (Arg-1), insulin-like growth factor-1 (IGF-1), chitinase-3-like protein 3 (Chi3l3/Ym-1), and found in the inflammatory zone (FIZZ) (Cao and He, 2013; Caldeira et al., 2017; Salvi et al., 2017; Zhou T. et al., 2017). Simultaneous down-regulation of M1 and up-regulation of the M2 phenotype in post-stroke conditions can be beneficial in minimizing the post-stroke insults.

Histone 3 lysine acetylation (H3KAc) is up-regulated in microglia around the peri-infarct and infarct zone after ischemic stroke. Similar up-regulation in H3KAc was also noted in lipopolysaccharide (LPS) mediated microglial activation. Thus, H3KAc up-regulation is highly associated with inflammatory cytokines. Histone deacetylase (HDAC) is a key regulator of H3KAc (Demyanenko et al., 2020; Fessler et al., 2013; Kong et al., 2018). HDAC inhibition promotes the downregulation of pro-inflammatory genes, such as TNF-α, iNOS, signal transducer and activator of transcription 1 (STAT1), and IL-6, and up-regulation of interleukin-10 (IL-10) and signal transducer and activator of transcription (STAT3) genes in activated microglia, both *in vivo* and *vitro*. The up-regulation of anti-inflammatory genes promotes neuronal survival, reduction in brain infarct volume, and suppression of microglia activation (M1) which shows the neuroprotective abilities of HDAC inhibitors (Kim et al., 2007; Patnala et al., 2017). HDAC6, as an adaptor, can affect aggrephagy in CNS. For instance, HDAC6-mediated aggregation is associated with retrograde axonal transport (Xu et al., 2021). Suberoylanilide hydroxamic acid (SAHA), which is an HDAC inhibitor, has been exhibited to up-regulate 70 kilodalton heat shock protein (Hsp70; essential for protein folding and stress-related protection in cells) and B-cell lymphoma 2 (Bcl-2; anti-apoptotic) along with the reduction of pro-inflammatory cytokines, thus preventing neuronal loss and promoting favorable outcome in post-stroke condition (Faraco et al., 2006; Langley et al., 2009; Abend and Kehat, 2015; Jhelum et al., 2017).

Apart from SAHA, other HDAC inhibitors such as valproic acid (VPA), sodium butyrate (SB), trichostatin-A (TSA), and sodium 4-phenylbutyrate (4-PBA) have been shown to promote similar neuroprotective abilities by regulation of excitotoxicity, oxidative stress, endoplasmic reticulum stress (ER-stress), apoptosis, inflammation, and BBB breakdown (Fessler et al., 2013). Reactive oxygen species (ROS) have a well-established association with cerebrovascular accidents (Olmez and Ozyurt, 2012; Qu et al., 2016). Nuclear factor erythroid 2-related factor 2 (Nrf-2) has been identified as a key regulator in ROS-dependent oxidative insults to CNS (Li et al., 2011; Yamauchi et al., 2016). Up-regulation of Nrf-2 using HDAC inhibitors such as VPA and TSA has been exemplified to promote neuroprotection against oxidative stress (Correa et al., 2011; Fessler et al., 2013).

Histone methylation has also been extensively explored to determine factors associated with prognostic outcomes in both pre and post-stroke conditions. Aging is one of the principal determinants of functional outcomes in cerebrovascular accidents (Manwani et al., 2011; Zhang et al., 2018) and is highly associated with a reduction in brain plasticity (Guggisberg et al., 2019; Nesin et al., 2019). A murine study revealed a significant reduction of Trimethylation of Histone H3 at lysine 4 (H3K4me3) in cortical astrocytes with progression in age (Chisholm et al., 2015). Histone 3 lysine 9 (H3K9) has also been identified as a potential target therapy region as inhibition of Histone-lysine N-methyltransferase SUV39H1 and Euchromatic histone-lysine N-methyltransferase 2 (G9a) promotes up-regulation of brain-derived neurotrophic factor (BDNF) in E17 neuronal cells (Schweizer et al., 2015). Another study using dimethyloxalylglycine (DMOG) to inhibit histone lysine demethylase subfamily 4 (KDM4) has been shown to promote neuronal repair via H3K9me2 dependent manner in CD1 mice (Chakravarty et al., 2017).

**FIGURE 3:**
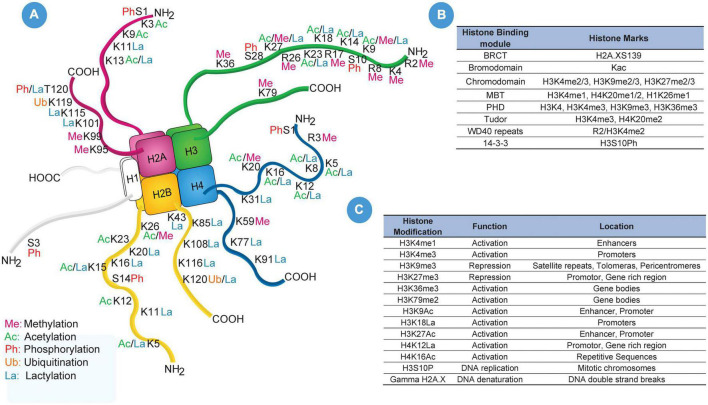
Histone Modifications. **(A)** Illustrates pictorial representation of all known till date post-translational modification of histone amino-terminal tail and their location regions. **(B)** Portraits frequency used histone marks and their histone binding modules such as BRCT, bromodomain, chromodomain, MBT, PHD, Tudor, WD40 repeats and 14-3-3. **(C)** List of frequently used histone-modified regions, functions, and locations in DNA sequence which includes H3K4me1, H3K4me3, H3K9me3, H3K27me3, H3K36me3, H3K79me2, H3K9Ac, H3K9La, H3K27Ac, H4K12La, H4K16Ac, H3S10P, and Gamma H2A.X.

Apart from histone acetylation and methylation, post-translational phosphorylation has also been identified in cerebral ischemic conditions (Crowe et al., 2006; Song et al., 2010; Liu et al., 2014; Zhao et al., 2016). Crowe SL and colleagues demonstrated an increase in ionotropic glutamate receptor (NMDA) activity that promotes histone phosphorylation (γ-H2A.X) in rat cortical neurons. However, pretreatment with vitamin E and BAPTA-AM (calcium chelator) attenuated γ-H2A.X formation (Crowe et al., 2006). A study using the Drosophila model demonstrated neuronal necrosis through phosphorylation of histone 3 serine 28 (H3S28Ph) (Liu et al., 2014). A list of commonly undertaken histone modification and histone binding modules has been enlisted in Figures 3A–C.

## DNA methylation

DNA methylation has been one of the most extensively studied epigenetic modifications, exclusively occurring at CpG dinucleotides in mammals and always symmetrical to maintain the methylation during the cell division process. Notably, DNA methylation depends on the balance between hyper and hypomethylation activity. DNA methylation is carried out by *de novo* methyltransferases (DNMT); precisely DNMT3a and DNMT3b in mammals. CpGs are clustered into CpG islands, often at the promotor site of the gene. CpG island tends to be protected from methylation. Methylation observed at CpG island is entirely associated with the silencing of gene expression and carried out either by the formation of repressive chromatin structure or inhibiting transcription factor binding and alteration of gene expression.

Long interspersed nuclear element-1 (LINE-1), which is a class I transposable element in the DNA and a member of LINEs has been the center of many study discussions after their discovery concerning the association in predicting increased risk of ischemic stroke and cardiovascular events. Hypomethylation of LINE-1 is associated with an increased risk of ischemic stroke (Baccarelli et al., 2010b; Lin et al., 2014; Soriano-Tarraga et al., 2014; Ng et al., 2018). However, a single sex-specific analytic study has demonstrated that LINE-1 hypomethylation is suggestive of advanced atherosclerotic lesions, which leads to global hypomethylation and has more association in determining the risk of development of ischemic stroke in men as compared to that of women (Lin et al., 2014). A further investigation reported the co-relation between hypomethylation of LINE-1 and an increased level of circulating vascular cell adhesion molecule-1 (VCAM-1) (Baccarelli et al., 2010a).

A cross-sectional study was conducted on the Japanese population aiming to determine the relationship between methylation of LINE-1 in leukocytes and that dyslipidemia. Hypomethylation of LINE-1 in leukocytes was showcased to have a higher odds ratio in individuals with dyslipidemia (Tsuboi et al., 2018). Thus, the methylation status of LINE-1 can be a key risk factor predictor. Similarly, hypomethylation of TNF receptor-associated factor 3 (TRAF3) and hypermethylation of thrombospondin-1 (THBS1) has also been illustrated to be crucial predictor of stroke-related outcomes (Lopez-Dee et al., 2011; Udali et al., 2013; Gallego-Fabrega et al., 2016; Ng et al., 2018). DNMT, especially DNMT1 and DNMT3a has also been identified as pivotal enzymes regulating methylation of various genes (Feng et al., 2010; Wu et al., 2012; Gustafsson et al., 2018), of which DNMT1-dependent DNA methylation has been pinpointed as a mediator of chronic inflammation and development of atherosclerotic disease via the peroxisome proliferator-activated receptor gamma (PPAR-γ) pathway (Yu et al., 2016). On the other hand, DNMT3a has also been identified to promote ischemic brain damage (Morita et al., 2013; Pandi et al., 2013). Thus, DNA hypomethylation may be a potential therapeutic strategy for the treatment of stroke (Sharifulina et al., 2021).

Matrix metalloproteinase-2 (MMP-2) is one of the most studied enzymes concerning their changes in peripheral blood concentration both in acute and chronic phases of post-stroke symptoms (Fatar et al., 2008; Kreisel et al., 2012, 2016). However, various studies have produced not identical data, creating confusion within the research field. A study conducted over a sample size of 556 participants (298 with ischemic stroke versus 258 control) successfully showcased a lower concentration of MMP-2 methylation level in peripheral blood exclusively in male small-vessel occlusion participants (Lin et al., 2017). Thus, narrowing the use of MMP-2 serum concentration as an effective marker in post-ischemic stroke. Apart from the common methylation at the fifth position of the pyrimidine ring of cytosine (5mC), other forms of modifications are also noted at a similar position namely, 5-hydroxymethyl (5hmC), 5-formal (5fC), and 5-carboxyl (5caC). Various studies have successfully showcased 5-hmC to regulate several cellular processes which include neuronal development as well. A neoteric study was conducted in murine specie (mouse), demonstrating the use of ascorbate (mineral salt of ascorbic acid; vitamin C) in post-stroke reperfusion led to Ten-eleven translocation 3 (TET3) dependent conversion of 5mC to 5hmC, promoting up-regulation of neuroprotective genes and functional recovery (Morris-Blanco et al., 2019).

5-aza-2′-deoxycytidine which is a DNA methyltransferase inhibitor (DNA methylation inhibitor) has been illustrated to significantly reduce the infarct volume (Endres et al., 2000). Likewise, another study using zebularine, which is also a DNA methylation inhibitor, has demonstrated dose-dependent (500 μg and 100 μg) reduction in infarct volume (Dock et al., 2015).

## RNA modification

**FIGURE 4:**
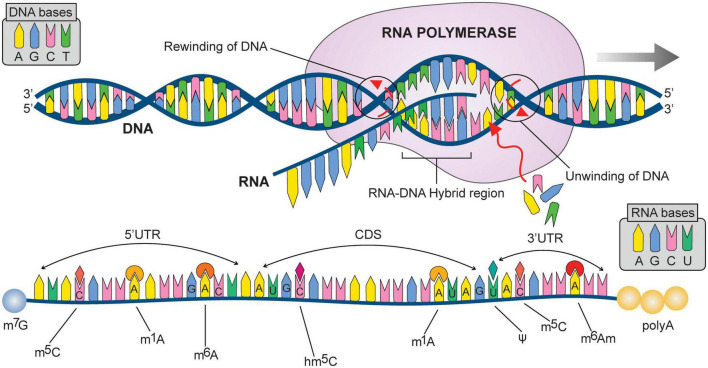
Illustrate DNA to RNA transcription (followed by possible mRNA (messenger RNA) modifications at different nitrogen bases). N6-methyladenosine m^6^A; Pseudouridine (Ψ); 5-methylcytosine (m^6^C); 5-hydroxymethylcytidine (hm^6^C); N1-methyladenosine (m1A); 7-Methylguanosine (m^7^G); N6,2′-O-dimethyladenosine (m^6^Am). 3′UTR: Three prime untranslated regions; CDS: CoDing Sequence; 5′UTR: Five prime untranslated regions.

Similar to DNA modifications, RNA modifications have also been shown to be a regulator of gene expression (Li et al., 2017; Engel and Chen, 2018; Coker et al., 2019; Sendinc et al., 2019). To date, RNA modifications include N^6^-methyladenosine (m^6^A), N6,2′-O-dimethyladenosine (m^6^Am), N1-methyladenosine (m^1^A), 5-methylcytosine (m^5^C), 5-hydroxymethylcytosine (hm^5^C), N4-acetylcytidine (ac^4^C), rotation isomerization of uridine/pseudouridine (ψ) and 7-Methylguanosine (m^7^G) (Figure 4). m^6^A is one of the most commonly observed mRNA modifications (Ji et al., 2018) and was identified in the 1970s (Desrosiers et al., 1974; Adams and Cory, 1975; Aloni et al., 1979). However, their association with small nuclear RNAs (snRNAs), micro-RNAs (miRNAs) circular RNA (circRNAs), and long non-coding RNAs (lncRNAs) has been recently understood (Dominissini et al., 2012; Chen et al., 2020). Mapping of m^6^A over human and murine RNA has identified over 18,000 m^6^A sites in 7,000 human genes with a consensus sequence of [G/A/U][G > A] m^6^A[U > A/C] (Dominissini et al., 2012; Meyer et al., 2012; Sun et al., 2016). m^6^A has also been shown to be changed during embryonic brain development and cerebral ischemic conditions (Meyer et al., 2012; Li et al., 2022). Furthermore, the silencing of m^6^A methyltransferase affects gene expression and modulates the p53 (TRP53) signaling pathway and apoptosis (Dominissini et al., 2012). Likewise, m^6^Am, m^1^A, m^5^C, hm^5^C, ac^4,^ C, ψ, and m^7^G are somewhat understood in the context of cancer and as potential biomarkers. For example, m^1^A was identified as a modulator in cerebral ischemic stroke (Chokkalla et al., 2022), and m^6^A was showcased to regulate the brain functions, development of synaptic plasticity, and their association with neuropsychiatric disorders (Yoon et al., 2017).

Cumulatively, epigenetic mechanisms offer a promising new therapeutic target in ischemia (Table 1). Histone/DNA/RNA modifications have been widely studied over the last decade. However, their contributions to stroke pathophysiological processes (including hemorrhagic and ischemic stroke) are still limited. Further clinical studies should assess whether these targets can restore or enhance significantly clinical outcomes of stroke patients.

**TABLE 1:** Potential molecules and their targets on epigenetics in ischemic stroke.

Name	Target	Species	Cell type	Model	Mechanism of action	References
SAHA	Histone	C57BL/6 mice	Neuron/Microglia/ Astrocyte	MCAO	Hsp70 and Bcl-2	Abend and Kehat, 2015; Faraco et al., 2006; Jhelum et al., 2017; Langley et al., 2009
SB	Histone	C57BL/6 mice	Microglia	MCAO	IL-10/STAT3	Patnala et al., 2017
4-PBA	Histone	C57BL/6 mice	Neuron	Hypoxia	HDAC	Qi et al., 2004
Chaetocin	Histone	Rats	Neuron	OGD	BDNF	Schweizer et al., 2015
DMOG	Histone	CD1 mouse	Neuron	ICAO	KMTs/KDMs	Chakravarty et al., 2017
JIL-1/MSK	Histone (H3S28ph)	Drosophila and C57BL/6 mice	Neuron	MCAO	PRC1/Trx	Liu et al., 2014
LINE-1	DNA	Human	–	AIS patients	Methylation	Lin et al., 2014
Clopidogrel	DNA	Human	–	AIS patients	TRAF3	Gallego-Fabrega et al., 2016
DNMT1	DNA	C57BL/6 embryos and Human	Macrophage	AIS	PPAR-γ	Yu et al., 2016
MMP-2	DNA	Human	–	AIS patients	Methylation	Lin et al., 2017
Ascorbate	DNA	C57BL/6 mice	Neuron/Astrocyte	MCAO	TET3/5hmC	Morris-Blanco et al., 2019
5-aza-2′-deoxycytidine	DNA	Transgenic mice	Neuron	MCAO	Methylation	Endres et al., 2000
Zebularine	DNA	Rats	Astrocyte	MCAO	Methylation	Chisholm et al., 2015
m^1^A	RNA	C57BL/6 mice	–	MCAO	m^1^A demethylase.	Chokkalla et al., 2022
m ^6^ A	RNA	C57BL/6 mice	–	MCAO	RNA methylation	Li et al., 2022

4-PBA: Sodium 4-phenylbutyrate; 5hmC: 5-hydroxymethyl; AIS: Acute ischemic stroke; Bcl-2: B-cell lymphoma 2; BDNF: Brain-derived neurotrophic factor; DMOG: dimethyloxalylglycine; DNMT 1: *de novo* methyltransferases 1; HDAC: Histone deacetylases; Hsp70: 70 kilodalton heat shock proteins; ICAO: Internal carotid artery occlusion; IL-10: Interleukin-10; JIL-1: Chromosomal serine/threonine-protein kinase-1; KMTs: Lysine methyltransferases; KDMs: Lysine demethylases; LINE-1: Long interspersed nuclear element-1; m1A: N1-methyladenosine; m6A: N6-methyladenosine; MCAO: Middle cerebral artery occlusion; MMP-2: Matrix metalloproteinase-2; MSK: Mitogen- and stress-activated kinase; OGD: Oxygen-glucose deprivation; PPAR-γ: Peroxisome proliferator-activated receptor gamma; PRC1: Polycomb repressive complex 1; SAHA: Suberoylanilide hydroxamic acid; SB: Sodium butyrate; STAT3: Signal transducer and activator of transcription 3; TET3: Tet methylcytosine dioxygenase 3; TRAF3: TNF receptor-associated factor 3: Trx: Thioredoxin; TSA: Trichostatin-A; VPA: Valproic acid.

## Prospect

As a result of interventions in the hyperacute phase, the mortality of stroke has declined substantially. However, long-term disability and institutionalization of the post-stroke remain unchanged. Stroke is a complex, multifactorial disease in which a wide plethora of pathological processes are simultaneously set in motion. Modulation of a single molecular factor is unlikely to be sufficient to attenuate or reverse the progression of stroke pathology. Epigenetic alterations such as DNA methylation, histone modifications, and RNA modifications are potent modulators of gene regulation, and an accumulating body of evidence suggests that they play a pivotal role in regulating brain remodeling after stroke. As a result, efforts are being made to identify key molecular signatures and development of combination therapy strategies similar to cancer (Dawson and Kouzarides, 2012).

Specifically, DNA methylation has been one of the heavily researched topics over the last decade and their association with risk factor prediction has been well documented. For example, DNA methylation of Cyclin-dependent kinase inhibitor 2B (CDKN2B) has been showcased to promote an increased risk of arterial calcification in ischemic stroke patients (Zhou et al., 2016; Zhou S. et al., 2017). Similarly, histone modifications have been illustrated to be a regulator of gene expression (Crowe et al., 2006; Kim et al., 2007; Schweizer et al., 2015; Patnala et al., 2017). Furthermore, strokes could cause an increase in anaerobic metabolism and lactic acidosis. Recently, a novel function for lactate is utilized in a new histone modification, histone lysine lactylation (Zhang et al., 2019; Figure 2). Pan et al. report an H4K12 lactylation positive feedback loop in microglial inflammation (Pan et al., 2022). This epigenetic mechanism may bring forth new biology and functionality to the role of metabolic homeostasis in regulating the secondary brain after stroke. The antagomir approach has been proven to promote neuroprotective effects in animal models of stroke and potential treatment strategies for the subsequent trend in epigenetics. Pharmacological inhibitors of these epigenetic modifications have been studied in animal models of stroke (Tang et al., 2017) and are readily available as treatment options in the clinic (Santini et al., 2013).

Over the years, several clinical studies or clinical trials were conducted to determine effective treatment after hemorrhagic and ischemic strokes. As mentioned earlier, HDAC inhibition could promote the downregulation of pro-inflammatory genes. VPA, a nitrogen-free broad-spectrum antiepileptic compound, has been used clinically for decades due to its effect on the decrease in neuronal hyperexcitability both by strengthening GABAergic transmission and by inhibiting sodium/especially calcium ion channels and HDACs. Previous clinical trials (Trial No. NCT01115959) reported that VPA-treated ICH patients had improved the National Institute of health stroke scale (NIHSS) scores (Gilad et al., 2011; Brookes et al., 2018). An ongoing study (Trial No. ChiCTR2100050161) also focuses on the effects of sodium valproate in patients with SAH (Chen et al., 2022). Other histone modification-related drugs, including Fluoxetine and Sildenafil citrate, are reported in clinical trials for both hemorrhagic and ischemic stroke (Chollet et al., 2011; Washington et al., 2016; Dennis et al., 2020; Marquez-Romero et al., 2020). Although clinical studies (Trial No. ISRCTN83290762, NCT00657163, NCT01737541) reported that Fluoxetine did not improve patients’ functional outcomes, early prescription of Fluoxetine with physiotherapy enhanced motor recovery in AIS and ICH patients (Chollet et al., 2011; Dennis et al., 2020; Marquez-Romero et al., 2020). Other epigenetic therapies, such as D-cycloserine (Trial No. NCT02082912), intra-arterial autologous bone marrow mononuclear cells injection (RNA modification, Trial No. NCT02178657), intravenous transplantation of autologous mesenchymal stem cells expanded with autologous serum (involved in non-coding RNA functions, Trial No. NCT01716481) also showed beneficial effects in ischemic stroke patients (Butler et al., 2015; Mancha et al., 2020; Bang et al., 2022) (Table 2).

**TABLE 2:** Clinical trials with epigenetic-related agents in stroke research.

Agent	Disease	Trial no.	Country	Duration	Proposed mechanism	Intervention	Status	Final verdict	References
VPA	ICH	NCT01115959	Israel	Feb 2003 – Dec 2008	Blocking voltage-gated ion channels/Inhibiting histone deacetylase	Orally 400 mg twice daily for one month	Completed	VPA-treated patients had improved NIHSS scores	Gilad et al., 2011; Brookes et al., 2018
	SAH	ChiCTR21000 50161	China	Aug 2021 – Present		20 mg/kg daily intravenously for 7 days	Ongoing	–	Chen et al., 2022
Fluoxetine	AIS	ISRCTN8329 0762	UK	May 2015 – Oct 2021	Selective serotonin reuptake inhibitor/Histone deacetylase	20 mg once daily or matching placebo capsules for 6 months	Completed	Fluoxetine did not improve functional outcomes but decreased the occurrence of depression	Dennis et al., 2020
		NCT00657163	France	Mar 2005 – Dec 2010		20 mg daily for 3 months	Completed	Fluoxetine enhanced motor recovery after 3 months	Chollet et al., 2011
	ICH	NCT01737541	Mexico	Nov 2012 – Aug 2014			Completed	Fluoxetine was safe and helped to increase motor recovery 90 days after ICH.	Marquez-Romero et al., 2020
D-cycloserine	IS	NCT02082912	USA	Jun 2010 – Apr 2012	NMDA agonist	100 mg PO twice weekly for three weeks	Completed	D-cycloserine can’t provide greater gains in learning for stroke survivors	Butler et al., 2015
Sildenafil citrate	IS	NCT02628847	USA	Mar 2012 – Oct 2016	PDE5 inhibitor/Histone deacetylase	25 mg once per day for 14 days starting day 5-9 post stroke	Completed	Assessment of upper extremity and lower extremity motor impairment	–
	SAH	NCT03028298	USA	Dec 2016 – Present		20mg oral and 10mg intravenous; 60mg oral and 30 mg intravenous	Ongoing	–	Washington et al., 2016
BM-MNCs	AIS	NCT02178657	Spain	Ap 2015 – Oct 2021	RNA modification	Intra-arterial autologous BM-MNCs injection (dose 2 × 10^6^ per kilogram)	Completed	BM-MNC is related to precursor cell migration in stroke and smaller infarct volumes	(Mancha et al., 2020)
MSCs	AIS	NCT01716481	South Korea	Nov 2012 – Dec 2017	miRNAs	Intravenous transplantation of autologous MSCs	Completed	MSCs are correlated with improvement in motor function and MRI indices of plasticity	Bang et al., 2022

AIS: Acute ischemic stroke; BM-MNCs: Bone marrow mononuclear cells; ICH: Intracerebral hemorrhage; IS: ischemic stroke; miRNAs: micro-RNAs; MRI: magnetic resonance imaging; MSCs: Mesenchymal stem cells; NIHSS: National institute of health stroke scale; PDE5: Phosphodiesterase 5; SAH: Subarachnoid hemorrhage; VPA: Valproic acid.

Overall, previous studies have successfully demonstrated that stroke leads to epigenetic dysregulation which in turn triggers a series of cascade changes that cause neuroinflammation, oxidative stress, apoptosis, and several other secondary injury events. Other epigenetic modifications (such as acetylation, phosphorylation, and lactylation) and epigenetic regulators (such as lncRNAs, circRNAs, and miRNAs), although not discussed in this review, were also reported as translational targets in stroke research. Agents targeting epigenetic regulation are under development and entering clinical trials. Epigenetic modifications, such as methylation or non-coding RNA expression levels, may play a crucial role in antiplatelet treatment for stroke patients (Danielak et al., 2022). Regulation of these key triggers would be beneficial to produce the desired outcome in post-stroke conditions.

## Conclusion

Advancements in epigenetics research have led us to further understand the mechanisms of secondary injury. Future understanding of the key modulators at the molecular level and combination therapies would be new management strategies in post-stroke conditions.

## Author contributions

JPe conceived the entire review project, conceptualization, and literature search. FZ, LY, JW, and JPa drafted the figures. JPe, LZ, SY, and YJ wrote the manuscript. YJ overviewed and guided the conception of the entire project. All the authors contributed to the critical revision of the final manuscript.
